# Prevalence, awareness, and control of hypertension and associated factors among Royal Thai Army personnel in Thailand from 2017 to 2021

**DOI:** 10.1038/s41598-023-34023-z

**Published:** 2023-04-28

**Authors:** Boonsub Sakboonyarat, Jaturon Poovieng, Phutsapong Srisawat, Panadda Hatthachote, Mathirut Mungthin, Ram Rangsin, Kanlaya Jongcherdchootrakul

**Affiliations:** 1 Department of Military and Community Medicine, Phramongkutklao College of Medicine, Bangkok, 10400 Thailand; 2 Department of Medicine, Phramongkutklao College of Medicine, Bangkok, 10400 Thailand; 3 Department of Physiology, Phramongkutklao College of Medicine, Bangkok, 10400 Thailand; 4 Department of Parasitology, Phramongkutklao College of Medicine, Bangkok, 10400 Thailand

**Keywords:** Public health, Cardiovascular diseases, Epidemiology, Risk factors

## Abstract

Hypertension (HTN) is a potential risk factor for cardiovascular diseases. We aimed to determine the prevalence, awareness, and control of HTN among RTA personnel in Thailand. We conducted a series of cross-sectional studies from 2017 to 2021. HTN was defined by systolic blood pressure (BP) ≥ 140 mmHg or a diastolic BP ≥ 90 mmHg from a physical health examination, a history of HTN diagnosed by medical personnel, or taking antihypertensive medication. A total of 504,484 participants were included in the present study. The overall HTN prevalence was 29.4%. The prevalence of HTN among males was 30.5%, while it was 17.1% among females. Of the RTA personnel with HTN, 35.9% were aware of their condition. The overall control of HTN among RTA personnel with HTN was 15.8% in 2017 and 17.6% in 2021. Behavioral factors associated with HTN were current smoking, alcohol consumption, and sedentary behavior. A higher BMI was associated with higher HTN prevalence and HTN awareness but less likely to have controllable HTN. Male participants, younger individuals, current alcohol use, and sedentary behavior were associated with a lower prevalence of HTN awareness and controlled HTN. Current tobacco use was also associated with a lower prevalence of HTN awareness.

## Introduction

Hypertension (HTN) is a potential risk factor for cardiovascular diseases^1,2^. The global population of individuals aged 30 to 79 years with HTN doubled from 1990 to 2019, from 331 million women and 317 million men in 1990 to 626 million women and 652 million men in 2019^3^. Worldwide, 31.1% of adults have HTN, and the estimated HTN prevalence among females and males is 30.1% and 31.9%, respectively^4^. The control rates among people with HTN in 2019 were 23% and 18% for women and men, respectively^3^. HTN is a major modifiable risk factor for atherosclerotic cardiovascular diseases (ASCVD)^5,6^. Furthermore, uncontrolled HTN is a significant health problem leading to serious adverse events, including heart diseases, stroke and kidney diseases^7–9^.

The National Health Examination Survey in Thailand (NHES) V 2014 and the NHES VI 2019 data indicated that HTN affects approximately 25% of Thai adults^10,11^. The information on uncontrolled HTN among HTN patients visiting clinics in Bangkok, private hospitals and public hospitals of the Ministry of Public Health in Thailand between 2014 and 2015 demonstrated that the prevalence of uncontrolled HTN was 25.6%^12^, whereas that among HTN patients in a remote rural community in Thailand was 54.4%^13^.

According to the increasing burden of NCDs in Thailand, the Royal Thai Government has included the diagnosis, treatment, and laboratory testing for HTN in the Universal Health Coverage (UHC) benefits package since 2002^14^. HTN is a common health issue among Thai civilians; however, limited information is available regarding HTN among the Royal Thai Army (RTA) personnel. Regarding healthcare in the RTA, 37 hospitals under the RTA nationwide provide care in cooperation with the RTA Medical Department (RTAMED). Nationwide, approximately 130,000 RTA personnel are eligible to participate in yearly health examinations provided by the RTAMED. It was found that the behavioral risk for HTN among RTA personnel was relatively high compared with Thai civilians^15,16^. Therefore, the investigators aimed to determine the prevalence, awareness, and control of HTN and their associated factors among RTA personnel in Thailand from 2017 to 2021.

## Methods

### Study design and subjects

We conducted five annual cross-sectional studies from 2017 to 2021. Details of the study were published by Sakboonyarat et al. elsewhere^16^. We retrieved the dataset from the RTA personnel database of routine physical health examinations after obtaining permission from the RTAMED in Bangkok, Thailand. The RTAMED provides annual health examinations for all active-duty RTA personnel through the Army Institute of Pathology, the Armed Forces Research Institute of Medical Sciences, and 37 RTA hospitals in four regions nationwide in Thailand. The health examination data are reported to the RTAMED in Bangkok^16^.

**Figure 1:**
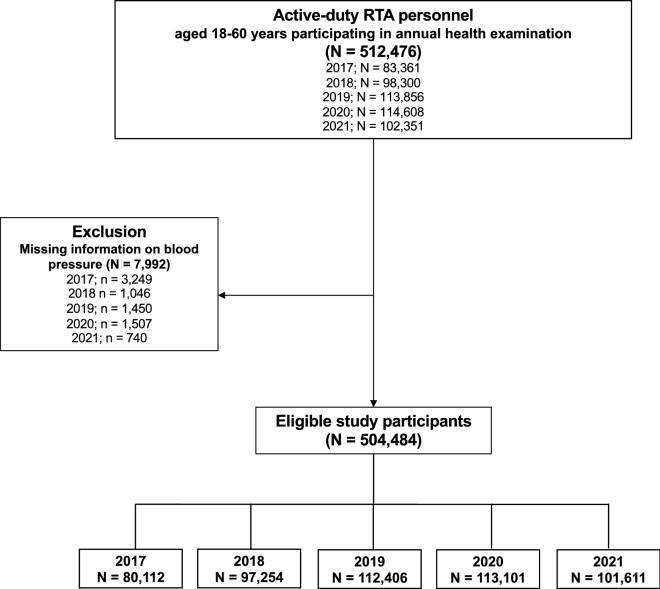
Flow chart of the study.

The sample size was calculated based on the prevalence of HTN among the Thai population from the NHES VI^11^, according to a type I error of 0.05. In the present study, the minimum calculated sample size was 7279 individuals^17^. In addition, to enhance the generalizability and represent the situation of HTN among the RTA personnel, we collected the data for all RTA personnel who participated in the annual health examination nationwide. The inclusion criteria for this study included active-duty RTA personnel aged 18–60 years nationwide. At the same time, participants who did not have information on blood pressure would be excluded from the analysis (Fig. 1).

### Data collection and measurement

Annually, the Army Institute of Pathology, the Armed Forces Research Institute of Medical Sciences and 37 RTA hospitals provide health examinations for RTA personnel. A self-report guide was created using a standardized case report form to obtain demographic characteristics, which comprised age, sex, regions, and health care schemes. In addition, information on comorbidities and behavioral risk factors was also collected, including a history of type 2 diabetes (T2D), smoking status, alcohol consumption and regular exercise.

Blood pressure (BP) was measured using an automatic blood pressure monitor, i.e., OMRON, HEM-7120, Kyoto, Japan, by an operator trained in the standardized technique following the Thai guidelines on the treatment of HTN. The participants were advised to avoid caffeine and smoking for at least 30 min before measurement. In addition, the participants were instructed to be stationary for at least five minutes in a chair, with their feet on the floor and their arms supported at heart level. Talking was not permitted during the measurement. Two measurements were taken, and the average was recorded^18^. The participants’ body weight was measured in kilograms, and the height was measured in centimeters.

A history of HTN and a history of T2D was defined using the data from the responses to the following questions: (1) “Have you ever been diagnosed with HTN or taken antihypertensive drugs?” and (2) “Have you ever been diagnosed with T2D or taken blood glucose lowering drugs?”. HTN was defined by a systolic blood pressure (SBP) ≥ 140 mmHg and/or a diastolic blood pressure (DBP) ≥ 90 mmHg and/or a history of HT diagnosed by medical personnel and/or a history of taking antihypertensive medication^18^. In the present study, participants were considered to be aware of HTN if they had a history of HTN diagnosed by medical personnel or taking antihypertensive drugs. Among those with HTN, controlled HTN was defined as an SBP < 140 mmHg and a DBP < 90 mmHg^19^.

Behavioral risk factors were obtained from the self-report questionnaire. Current smoking was defined as having smoked 100 cigarettes in his or her lifetime and who currently smokes cigarettes^21^. Current alcohol consumption was defined as having a history of consuming alcohol within 12 months^22^. Regular exercise was defined as an exercise for 30 min/day and at least three days/week^23^. Body mass index (BMI) was computed as body weight in kilograms divided by height (weight (kg)/height (m)^2^). We classified BMI into five groups, including < 18.50 kg/m^2^, 18.50–22.99 kg/m^2^, 23.00–24.99 kg/m^2^, 25.00–29.99 kg/m^2^, and ≥ 30.00 kg/m^2^^20^.

### Statistical analysis

Statistical analyses were performed using StataCorp. 2021 (*Stata Statistical Software: Release 17*. College Station, TX: StataCorp LLC). We calculated the frequency distribution of demographic characteristics, behavioral data and comorbidities to describe the study sample. Categorical variables, including sex, age group, region, health scheme, a history of T2D, smoking status, alcohol consumption, regular exercise and BMI categories, are presented as percentages. Continuous variables, including age, BMI, SBP and DBP, are presented as the mean and standard deviation (SD). Age- and sex-adjusted HTN prevalence, awareness, and control among RTA personnel were calculated and presented as percentages and 95% confidence intervals (CIs). The nonlinear trend was tested first by adding a quadratic term into the regression model. If the result was not significant, a linear trend was tested.

To determine the associations among categorical data, the *chi*-square test was used. Linear regression analysis was used to determine the association between the mean blood pressure and BMI categories. The logistic regression model was used to determine independent factors associated with HTN, awareness of HTN and control of HTN. In order to adjust the potential confounders, multivariable analysis was performed, which accounted for age, sex, regions, health insurance scheme, a history of diabetes, smoking status, alcohol use, exercise, BMI, and years. The magnitude of the association was presented as an adjusted odds ratio (AOR) with a 95% CI. A two-sided *p-*value less than 0.05 was considered statistically significant.

We also performed a sensitivity analysis to account for the nested data of the hospital that provided the health examination. We analyzed the factors associated with HT prevalence, awareness, and control using generalized linear mixed models with a logit link to adjust for potential confounders and including random effects to account for RTA hospital variability.

### Ethics consideration

The study was reviewed and approved by the Institutional Review Board, Royal Thai Army Medical Department (approval number S067h/64), in accordance with international guidelines, including the Declaration of Helsinki, the Belmont Report, CIOMS Guidelines, and the International Conference on Harmonization of Technical Requirements for Registration of Pharmaceuticals for Human Use-Good Clinical Practice (ICH-GCP). Due to using secondary data, a waiver of documentation of informed consent was used, and the waiver for informed consent was granted by the Institutional Review Board, Royal Thai Army Medical Department.

## Results

### Demographic characteristics

Table 1 shows the demographic characteristics of the 504,484 RTA personnel included in the study population between 2017 and 2021. The mean age of the participants ranged from 37.6 to 39.1 years. The majority (91.3%) were male. One-third (36.3%) of all participants resided in the central region. Almost all participants were under the civil servant medical benefit scheme (97.8%). The prevalence of current smoking was 28.5% in 2017 and rose to 33.4% in 2021. The average BMI of the participants has remained stable, ranging from 24.7 ± 3.7 kg/m^2^ to 25.0 ± 3.9 kg/m^2^ over 5 years. Table 2 presents the average SBP and DBP of the participants from 2017 to 2021. In 2017, the age- and sex-adjusted average SBP was 127.2 mmHg and slightly increased to 129.2 mmHg in 2021 (*p* for trend < 0.001), whereas the age- and sex-adjusted average DBP of the participants was 78.0 mmHg in 2017 and had remained stable at 78.2 mmHg in 2021 (*p* for trend = 0.262).

**Table 1:** Demographic characteristics of participants from 2017 to 2021 (N = 504,484).

Year	2017	2018	2019	2020	2021
Characteristics	N = 80,112	N = 97,254	N = 112,406	N = 113,101	N = 101,611
	n (%)	n (%)	n (%)	n (%)	n (%)
Sex					
Male	73,568 (91.8)	88,743 (91.2)	103,013 (91.6)	101,923 (90.1)	93,181 (91.7)
Female	6544 (8.2)	8511 (8.8)	9393 (8.4)	11,178 (9.9)	8430 (8.3)
Age (years)					
Mean ± SD	39.1 ± 11.5	38.2 ± 11.4	38.0 ± 11.4	38.2 ± 11.5	37.6 ± 11.1
< 30	22,076 (27.6)	28,893 (29.7)	34,100 (30.3)	33,623 (29.7)	30,160 (29.7)
30–39	21,068 (26.3)	26,929 (27.7)	31,929 (28.4)	33,184 (29.3)	32,732 (32.2)
40–49	15,377 (19.2)	18,299 (18.8)	20,478 (18.2)	20,396 (18.0)	18,449 (18.2)
50–59	21,301 (26.6)	22,395 (23.0)	25,389 (22.6)	24,740 (21.9)	19,672 (19.4)
60	290 (0.4)	738 (0.8)	510 (0.5)	1158 (1.0)	598 (0.6)
Regions					
Bangkok	10,960 (13.7)	15,139 (15.6)	17,171 (15.3)	17,893 (15.8)	8730 (8.6)
Central	27,646 (34.5)	35,767 (36.8)	37,536 (33.4)	41,627 (36.8)	40,295 (39.7)
Northeast	15,047 (18.8)	16,792 (17.3)	19,056 (17.0)	21,603 (19.1)	17,558 (17.3)
North	19,351 (24.2)	15,209 (15.6)	23,881 (21.2)	18,530 (16.4)	22,755 (22.4)
South	7108 (8.9)	14,347 (14.8)	14,762 (13.1)	13,448 (11.9)	12,273 (12.1)
Health insurance scheme					
Civil servant medical benefit	78,540 (98.0)	95,221 (97.9)	110,154 (98.0)	109,868 (97.1)	99,658 (98.1)
Social Security	1009 (1.3)	1196 (1.2)	1423 (1.3)	2391 (2.1)	1645 (1.6)
Universal Coverage	563 (0.7)	837 (0.9)	829 (0.7)	842 (0.7)	308 (0.3)
History of diabetes					
No	71,340 (89.1)	87,778 (90.3)	101,393 (90.2)	102,018 (90.2)	91,513 (90.1)
Yes	8772 (10.9)	9476 (9.7)	11,013 (9.8)	11,083 (9.8)	10,098 (9.9)
Current smoker					
No	56,540 (71.5)	65,535 (68.8)	74,588 (68.3)	71,371 (66.3)	67,571 (66.6)
Yes	22,532 (28.5)	29,712 (31.2)	34,585 (31.7)	36,308 (33.7)	33,825 (33.4)
Current alcohol use					
No	25,171 (32.1)	31,927 (33.7)	36,121 (32.3)	31,076 (28.8)	35,029 (34.6)
Yes	53,298 (67.9)	62,681 (66.3)	75,799 (67.7)	76,949 (71.2)	66,350 (65.4)
Exercise					
No	6535 (8.4)	6028 (6.5)	6674 (6.1)	6569 (6.0)	8766 (8.7)
Irregular exercise	23,917 (30.7)	30,889 (33.3)	30,632 (28.1)	39,094 (35.8)	39,151 (38.7)
Regular exercise	47,484 (60.9)	55,975 (60.3)	71,822 (65.8)	63,463 (58.2)	53,327 (52.7)
Body mass index (kg/m^2^)					
Mean ± SD	24.7 ± 3.7	24.8 ± 3.8	24.9 ± 3.8	24.9 ± 3.9	25.0 ± 3.9
18.50–22.99	25,158 (31.4)	30,372 (31.2)	34,608 (30.8)	35,001 (30.9)	30,472 (30.0)
< 18.50	1766 (2.2)	2007 (2.1)	2276 (2.0)	2345 (2.1)	2028 (2.0)
23.00–24.99	19,503 (24.3)	23,585 (24.3)	27,104 (24.1)	26,804 (23.7)	24,239 (23.9)
25.00–29.99	26,952 (33.6)	32,588 (33.5)	37,834 (33.7)	37,821 (33.4)	34,328 (33.8)
$\ge$ 30.00	6733 (8.4)	8702 (8.9)	10,584 (9.4)	11,130 (9.8)	10,544 (10.4)

*SD* standard deviation.

**Table 2:** Trends in age- and sex-adjusted mean blood pressure among RTA personnel from 2017–2021.

	Total	2017	2018	2019	2020	2021	*p* for trend^†^
	Mean ± SE	Mean ± SE	Mean ± SE	Mean ± SE	Mean ± SE	Mean ± SE	
Systolic blood pressure (mmHg)							
Overall^a^	128.3 ± 0.1	127.2 ± 0.1	128.0 ± 0.1	128.2 ± 0.1	128.5 ± 0.1	129.2 ± 0.1	< 0.001
Sex^b^							
Male	129.1 ± 0.1	127.9 ± 0.1	128.9 ± 0.1	129.1 ± 0.1	129.3 ± 0.1	130.0 ± 0.1	< 0.001
Female	119.5 ± 0.1	119.3 ± 0.2	119.0 ± 0.2	119.1 ± 0.1	119.9 ± 0.1	120.3 ± 0.2	< 0.001
Age^c^							
< 30	124.5 ± 0.1	123.0 ± 0.1	124.4 ± 0.1	124.7 ± 0.1	124.8 ± 0.1	125.1 ± 0.1	< 0.001
30–39	126.6 ± 0.1	125.3 ± 0.1	126.3 ± 0.1	126.5 ± 0.1	126.9 ± 0.1	127.4 ± 0.1	< 0.001
40–49	129.6 ± 0.1	128.6 ± 0.1	129.4 ± 0.1	129.4 ± 0.1	129.9 ± 0.1	130.8 ± 0.1	< 0.001
50–59	134.0 ± 0.1	133.4 ± 0.1	133.6 ± 0.1	133.8 ± 0.1	134.2 ± 0.1	135.4 ± 0.1	< 0.001
60	136.8 ± 0.3	138.2 ± 1.0	136.4 ± 0.6	136.1 ± 0.8	136.1 ± 0.5	138.6 ± 0.7	0.490‡
Regions^a^							
Bangkok	127.5 ± 0.1	129.4 ± 0.1	126.7 ± 0.1	127.4 ± 0.1	127.5 ± 0.1	126.4 ± 0.2	< 0.001
Central	129.5 ± 0.1	128.1 ± 0.1	129.7 ± 0.1	130.4 ± 0.1	129.4 ± 0.1	129.6 ± 0.1	0.001
Northeast	130.3 ± 0.1	128.4 ± 0.1	130.2 ± 0.1	128.9 ± 0.1	130.7 ± 0.1	133.3 ± 0.1	< 0.001
North	125.9 ± 0.1	124.3 ± 0.1	125.1 ± 0.1	125.5 ± 0.1	126.5 ± 0.1	127.6 ± 0.1	< 0.001
South	126.3 ± 0.1	125.2 ± 0.2	125.6 ± 0.1	126.8 ± 0.1	126.4 ± 0.1	126.8 ± 0.1	< 0.001
Diastolic blood pressure (mmHg)							
Overall^a^	78.1 ± 0.1	78.0 ± 0.1	78.3 ± 0.1	78.0 ± 0.1	78.0 ± 0.1	78.2 ± 0.1	0.262‡
Sex^b^							
Male	78.6 ± 0.1	78.5 ± 0.1	78.8 ± 0.1	78.5 ± 0.1	78.5 ± 0.1	78.6 ± 0.1	0.932
Female	73.3 ± 0.1	73.6 ± 0.1	73.6 ± 0.1	73.3 ± 0.1	72.9 ± 0.1	73.1 ± 0.1	< 0.001
Age^c^							
< 30	73.3 ± 0.1	73.2 ± 0.1	73.6 ± 0.1	73.3 ± 0.1	73.3 ± 0.1	72.9 ± 0.1	< 0.001
30–39	77.9 ± 0.1	77.8 ± 0.1	78.0 ± 0.1	77.8 ± 0.1	77.9 ± 0.1	77.9 ± 0.1	0.799‡
40–49	81.4 ± 0.1	81.1 ± 0.1	81.5 ± 0.1	81.2 ± 0.1	81.4 ± 0.1	81.8 ± 0.1	< 0.001
50–59	82.0 ± 0.1	82.2 ± 0.1	82.2 ± 0.1	81.8 ± 0.1	81.6 ± 0.1	82.2 ± 0.1	0.016
60	80.6 ± 0.3	81.3 ± 0.6	81.4 ± 0.4	80.6 ± 0.5	79.8 ± 0.3	81.2 ± 0.4	0.100‡
Regions^a^							
Bangkok	77.3 ± 0.1	78.3 ± 0.1	76.9 ± 0.1	77.3 ± 0.1	77.3 ± 0.1	76.4 ± 0.1	< 0.001
Central	78.4 ± 0.1	78.6 ± 0.1	79.1 ± 0.1	78.5 ± 0.1	78.1 ± 0.1	77.9 ± 0.1	< 0.001
Northeast	78.7 ± 0.1	78.4 ± 0.1	79.2 ± 0.1	77.8 ± 0.1	78.4 ± 0.1	79.7 ± 0.1	< 0.001
North	78.2 ± 0.1	77.1 ± 0.1	77.5 ± 0.1	78.3 ± 0.1	79.0 ± 0.1	78.9 ± 0.1	< 0.001
South	77.2 ± 0.1	77.4 ± 0.1	77.6 ± 0.1	77.4 ± 0.1	77.0 ± 0.1	76.5 ± 0.1	< 0.001

*SE* standard error.

^†^Nonlinear trend was tested first by adding a quadratic term into the regression model. If not significant, linear trend was tested.

^‡^Linear trend.

^a^Age- and sex-adjusted mean.

^b^Age-adjusted mean.

^c^Sex-adjusted mean.

### Prevalence of hypertension

**Figure 2:**
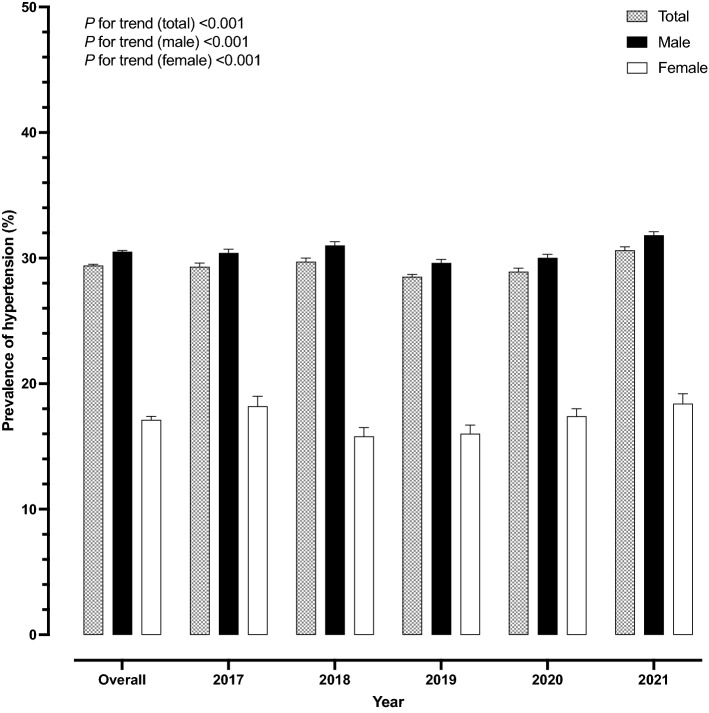
Age- and sex-adjusted prevalence of hypertension (%) and 95% CI among Royal Thai Army personnel from 2017 to 2021.

**Figure 3:**
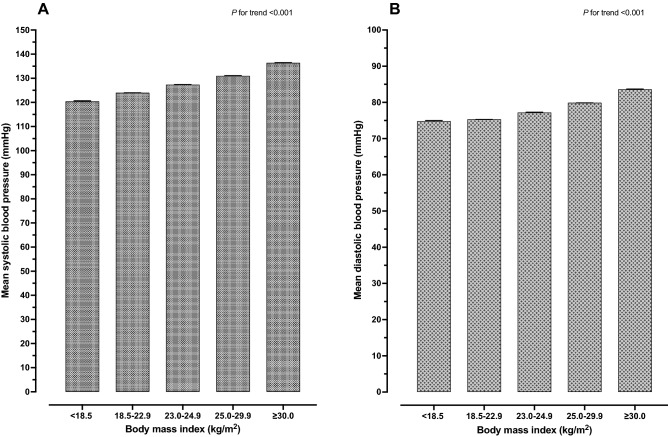
Average adjusted prediction of mean blood pressure (mmHg) and 95% CI, by body mass index categories. (**A**) Average adjusted prediction of mean systolic blood pressure among overall RTA personnel, adjusting for age, sex, regions, health insurance scheme, a history of diabetes, smoking status, alcohol use, exercise, and years. (**B**) Average adjusted prediction of mean diastolic blood pressure among overall RTA personnel, adjusting for age, sex, regions, health insurance scheme, a history of diabetes, smoking status, alcohol use, exercise, and years.

The prevalence of HTN among RTA personnel from 2017 to 2021 is summarized in Table 3. The age- and sex-adjusted HTN prevalence among RTA personnel was 29.3% in 2017, slightly dropped to 28.5% in 2019 and rose to 30.6% in 2021 (*p* for trend < 0.001). The overall HTN prevalence among males and females was 30.5% and 17.1%, respectively. Figure 2 presents the age- and sex-adjusted HTN prevalence among males and females from 2017 to 2021. In terms of age, HTN prevalence among RTA personnel under 30 years of age was 14.2% and incrementally increased, with a higher age group reaching a prevalence of 58.0% at 60 years of age. Regarding geographic region, the overall prevalence of HTN among RTA personnel residing in the northeast was 35.0%, which was the highest among the regions. In addition, rising trends in the prevalence of HTN among RTA personnel residing in the northeast were observed; it rose from 30.5% in 2017 to 40.3% in 2021 (Table 3). The higher the recorded BMI, the higher the prevalence of HTN observed. The prevalence of HTN among RTA personnel with a BMI of 18.50 to 22.99 kg/m^2^ was 17.0%, while it was 64.1% among individuals with a BMI $\ge$ 30.00 kg/m^2^ (Table 4). Figure 3 illustrates that the rising average SBP (A) and DBP (B) were significantly related to higher BMI (*p-value* < 0.001).

**Table 3:** Trends in age- and sex- adjusted prevalence, awareness, and control of hypertension among RTA personnel from 2017 to 2021.

	Total		2017		2018		2019		2020		2021		*p* for trend^†^
	n	% (95% CI)	n	% (95% CI)	n	% (95% CI)	n	% (95% CI)	n	% (95% CI)	n	% (95% CI)	
Hypertension													
Overall^a^	148,155	29.4 (29.2–29.5)	24,577	29.3 (29.0–29.6)	28,957	29.7 (29.4–30.0)	31,787	28.5 (28.2–28.7)	32,490	28.9 (28.7–29.2)	30,344	30.6 (30.4–30.9)	< 0.001
Sex^b^													
Male	140,631	30.5 (30.4–30.7)	23,356	30.4 (30.1–30.7)	27,528	31.0 (30.7–31.3)	30,258	29.6 (29.4–29.9)	30,576	30.0 (29.7–30.3)	28,913	31.8 (31.5–32.1)	< 0.001
Female	7524	17.1 (16.7–17.4)	1221	18.2 (17.3–19.0)	1429	15.8 (15.1–16.5)	1529	16.0 (15.3–16.7)	1914	17.4 (16.7–18.0)	1431	18.4 (17.7–19.2)	< 0.001
Age^c^													
< 30	21,072	14.2 (14.0–14.3)	3151	14.2 (13.8–14.7)	4509	15.5 (15.1–15.9)	4919	14.4 (14.0–14.7)	4452	13.4 (13.0–13.7)	4041	13.4 (13.0–13.8)	< 0.001
30–39	33,299	22.8 (22.6–23.0)	4828	22.9 (22.3–23.5)	6288	23.4 (22.9–23.9)	7050	22.0 (21.6–22.5)	7175	21.8 (21.3–22.2)	7958	24.2 (23.8–24.7)	0.001
40–49	33,937	36.5 (36.2–36.8)	5618	36.4 (35.6–37.1)	6714	36.7 (36.0–37.4)	7128	34.8 (34.1–35.4)	7242	35.8 (35.2–36.5)	7235	39.1 (38.4–39.8)	< 0.001
50–59	57,937	51.0 (50.8–51.3)	10,793	50.4 (49.8–51.1)	11,075	49.5 (48.9–50.2)	12,426	48.9 (48.3–49.5)	12,908	52.4 (51.7–53.0)	10,735	54.5 (53.8–55.2)	< 0.001
60	1910	58.0 (56.3–59.7)	187	65.4 (60.0–70.9)	371	50.8 (47.2–54.4)	264	52.1 (47.8–56.5)	713	61.2 (58.4–64.0)	375	62.0 (58.1–65.9)	< 0.001
Regions^a^													
Bangkok	17,434	24.9 (24.6–25.3)	3299	29.5 (28.7–30.2)	3551	22.2 (21.6–22.8)	3989	23.6 (23.0–24.2)	4491	25.6 (25.0–26.2)	2104	25.2 (24.4–26.1)	0.001^‡^
Central	55,846	30.5 (30.3–30.7)	9512	32.4 (31.9–32.9)	11,700	32.6 (32.2–33.1)	11,231	29.7 (29.3–30.1)	11,827	29.1 (28.7–29.5)	11,576	29.6 (29.2–30.0)	< 0.001
Northeast	31,529	35.0 (34.7–35.3)	4742	30.5 (29.8–31.2)	6006	36.2 (35.5–36.9)	6395	33.9 (33.2–34.5)	7408	34.0 (33.4–34.6)	6978	40.3 (39.6–41.0)	< 0.001
North	28,437	28.5 (28.2–28.8)	5223	26.2 (25.6–26.8)	4193	27.9 (27.2–28.6)	6688	28.1 (27.5–28.6)	5575	30.0 (29.4–30.6)	6758	30.2 (29.7–30.8)	< 0.001
South	14,909	24.1 (23.7–24.4)	1801	24.0 (23.0–24.9)	3507	24.4 (23.7–25.0)	3484	24.5 (23.8–25.1)	3189	23.8 (23.1–24.5)	2928	23.6 (22.9–24.3)	0.158^‡^
Awareness													
Overall^a^	53,129	35.9 (35.6–36.1)	9124	35.7 (35.2–36.3)	9844	34.7 (34.2–35.2)	11,047	35.3 (34.8–35.8)	11,995	36.0 (35.5–36.4)	11,119	37.6 (37.1–38.1)	< 0.001
Sex^b^													
Male	48,996	34.8 (34.6–35.1)	8440	34.7 (34.1–35.2)	9071	33.6 (33.1–34.2)	10,227	34.3 (33.9–34.8)	10,895	34.8 (34.3–35.3)	10,363	36.7 (36.2–37.2)	< 0.001
Female	4133	54.9 (53.8–56.1)	684	55.6 (53.0–58.3)	773	54.0 (51.6–56.5)	820	53.1 (50.7–55.5)	1100	56.7 (54.6–58.9)	756	54.8 (52.3–57.3)	0.631^‡^
Age^c^													
< 30	1666	7.9 (7.5–8.3)	255	8.1 (7.1–9.1)	502	11.1 (10.2–12.0)	460	9.4 (8.6–10.2)	189	4.2 (3.7–4.8)	260	6.4 (5.7–7.2)	< 0.001
30–39	5566	16.7 (16.3–17.1)	832	17.1 (16.1–18.2)	1030	16.4 (15.5–17.3)	1108	15.8 (15.0–16.7)	977	13.6 (12.8–14.4)	1619	20.4 (19.5–21.2)	< 0.001
40–49	13,242	39.0 (38.5–39.5)	2262	40.4 (39.1–41.6)	2396	35.8 (34.6–36.9)	2709	38.0 (36.9–39.1)	2827	38.9 (37.8–40.0)	3048	42.1 (41.0–43.3)	< 0.001
50–59	31,428	54.2 (53.8–54.7)	5646	52.4 (51.4–53.3)	5672	51.2 (50.3–52.2)	6608	53.2 (52.3–54.1)	7529	58.2 (57.4–59.1)	5973	55.7 (54.7–56.6)	< 0.001
60	1227	64.2 (62.1–66.4)	129	68.1 (61.3–74.9)	244	65.5 (60.7–70.4)	162	65.5 (60.7–70.4)	473	66.5 (63.1–70.0)	219	58.9 (53.9–63.8)	0.107^‡^
Regions^a^													
Bangkok	10,121	58.1 (57.3–58.8)	1675	52.8 (51.2–54.4)	2091	56.7 (55.1–58.2)	2318	58.5 (57.1–59.9)	2733	60.9 (59.6–62.2)	1304	61.8 (59.9–63.7)	< 0.001
Central	21,544	38.6 (38.2–39.0)	4134	42.5 (41.6–43.4)	3923	34.8 (34.0–35.6)	3773	34.2 (33.3–35.0)	4757	39.0 (38.1–39.8)	4957	42.9 (42.1–43.8)	< 0.001
Northeast	9865	31.3 (30.8–31.8)	1343	26.6 (25.4–27.7)	1991	34.3 (33.1–35.4)	2323	36.9 (35.7–38.0)	2147	27.9 (27.0–28.9)	2061	30.7 (29.6–31.7)	0.003
North	8560	30.1 (29.6–30.6)	1616	29.4 (28.3–30.5)	1098	27.7 (26.5–29.0)	1940	29.3 (28.3–30.3)	1617	29.5 (28.4–30.6)	2289	33.4 (32.3–34.4)	< 0.001
South	3039	20.4 (19.7–21.0)	356	18.1 (16.6–19.7)	741	20.9 (19.7–22.1)	693	20.5 (19.2–21.7)	741	22.9 (21.6–24.2)	508	18.4 (17.1–19.7)	0.510^‡^
Control													
Overall^a^	23,944	16.2 (16.0–16.3)	4009	15.8 (15.4–16.2)	4430	15.6 (15.2–16.0)	4802	15.4 (15.0–15.7)	5490	16.3 (16.0–16.7)	5213	17.6 (17.2–18.1)	< 0.001
Sex^b^													
Male	21,660	15.4 (15.2–15.6)	3647	15.1 (14.6–15.5)	4000	14.9 (14.4–15.3)	4355	14.6 (14.2–15.0)	4887	15.6 (15.2–15.9)	4771	16.9 (16.4–17.3)	< 0.001
Female	2284	30.4 (29.3–31.4)	362	29.5 (27.0–32.0)	430	30.0 (27.7–32.4)	447	29.0 (26.8–31.2)	603	31.2 (29.1–33.2)	442	31.8 (29.4–34.2)	0.631^‡^
Age^c^													
< 30	903	4.3 (4.0–4.6)	127	4.0 (3.4–4.7)	289	6.4 (5.7–7.1)	267	5.5 (4.8–6.1)	71	1.6 (1.2–2.0)	149	3.7 (3.1–4.2)	< 0.001
30–39	2453	7.4 (7.1–7.6)	353	7.2 (6.5–8.0)	440	7.0 (6.4–7.6)	434	6.2 (5.7–6.8)	364	5.0 (4.5–5.5)	862	10.8 (10.2–11.5)	< 0.001
40–49	5802	17.1 (16.7–17.5)	999	17.9 (16.9–18.9)	1073	16.0 (15.2–16.9)	1115	15.6 (14.8–16.5)	1222	16.7 (15.9–17.6)	1393	19.3 (18.4–20.2)	< 0.001
50–59	14,228	24.6 (24.2–24.9)	2470	23.0 (22.2–23.7)	2529	22.8 (22.1–23.6)	2911	23.4 (22.7–24.2)	3606	27.8 (27.0–28.6)	2712	25.3 (24.5–26.1)	< 0.001
60	558	29.2 (27.2–31.3)	60	30.5 (24–37.1)	99	26.3 (21.8–30.8)	75	28.2 (22.8–33.6)	227	32.2 (28.7–35.6)	97	26.5 (22.0–31.0)	0.107^‡^
Regions^a^													
Bangkok	4937	28.3 (27.6–29.0)	685	21.7 (20.3–23.1)	1005	27.1 (25.7–28.5)	1168	29.5 (28.1–30.9)	1351	30.0 (28.7–31.3)	728	34.6 (32.6–36.6)	< 0.001
Central	9586	17.2 (16.9–17.5)	1725	17.8 (17.0–18.5)	1634	14.6 (13.9–15.2)	1374	12.5 (11.9–13.1)	2282	18.5 (17.8–19.1)	2571	22.3 (21.6–23.0)	< 0.001
Northeast	4201	13.3 (12.9–13.7)	600	12.1 (11.2–13.0)	898	15.4 (14.5–16.3)	1128	17.8 (16.9–18.8)	833	10.9 (10.2–11.6)	742	10.9 (10.2–11.7)	0.003
North	4035	14.2 (13.8–14.6)	851	15.6 (14.6–16.5)	603	15.3 (14.2–16.3)	900	13.6 (12.8–14.4)	733	13.4 (12.5–14.3)	948	13.7 (12.9–14.5)	< 0.001
South	1185	7.9 (7.5–8.4)	148	7.6 (6.5–8.7)	290	8.2 (7.4–9.1)	232	6.8 (6.0–7.7)	291	8.9 (7.9–9.8)	224	8.1 (7.1–9.1)	0.510^‡^

*SBP* systolic blood pressure, *DBP* diastolic blood pressure, *95% CI* 95% confidence interval.

^†^Nonlinear trend was tested first by adding a quadratic term into the regression model. If not significant, linear trend was tested.

^‡^Linear trend.

^a^Age- and sex-adjusted prevalence.

^b^Age-adjusted prevalence.

^c^Sex-adjusted prevalence.

**Table 4:** Prevalence, awareness and control of hypertension among RTA personnel by characteristics (2017–2021).

Characteristics	Hypertension	*p-value*	Awareness	*p-value*	Control	*p-value*
	% (95% CI)		% (95% CI)		% (95% CI)	
Sex						
Male	30.5 (30.4–30.7)	< 0.001	34.8 (34.6–35.1)	< 0.001	15.4 (15.2–15.6)	< 0.001
Female	17.1 (16.7–17.4)		54.9 (53.8–56.1)		30.4 (29.3–31.4)	
Age (years)						
< 30	14.2 (14.0–14.3)	< 0.001	7.9 (7.5–8.3)	< 0.001	4.3 (4.0–4.6)	< 0.001
30–39	22.8 (22.6–23.0)		16.7 (16.3–17.1)		7.4 (7.1–7.7)	
40–49	36.5 (36.2–36.8)		39.0 (38.5–39.5)		17.1 (16.7–17.5)	
50–59	51.0 (50.8–51.3)		54.2 (53.8–54.7)		24.6 (24.2–24.9)	
60	58.0 (56.3–59.7)		64.2 (62.1–66.4)		29.2 (27.2–31.3)	
Regions						
Bangkok	24.9 (24.6–25.3)	< 0.001	58.1 (57.3–58.8)	< 0.001	28.3 (27.7–29.0)	< 0.001
Central	30.5 (30.3–30.7)		38.6 (38.2–39.0)		17.2 (16.9–17.5)	
Northeast	35.0 (34.7–35.3)		31.3 (30.8–31.8)		13.3 (13.0–13.7)	
North	28.5 (28.2–28.8)		30.1 (29.6–30.6)		14.2 (13.8–14.6)	
South	24.1 (23.7–24.4)		20.4 (19.7–21.0)		7.9 (7.5–8.4)	
Scheme						
Civil servant medical benefit	29.6 (29.4–29.7)	< 0.001	35.8 (35.6–36.1)	< 0.001	16.1 (15.9–16.3)	< 0.001
Social Security	20.1 (19.2–21.0)		44.9 (42.4–47.4)		21.5 (19.5–23.6)	
Universal Coverage	21.0 (19.7–22.4)		23.8 (20.8–27.0)		11.5 (9.4–14.1)	
History of diabetes						
No	24.0 (23.9–24.2)	< 0.001	19.9 (19.7–20.2)	< 0.001	8.2 (8.0–8.4)	< 0.001
Yes	77.4 (77.1–77.8)		80.4 (80.0–80.8)		38.4 (37.9–38.9)	
Current smoker						
No	29.7 (29.5–29.8)	< 0.001	39.2 (38.9–39.5)	< 0.001	19.9 (19.5–20.3)	< 0.001
Yes	28.8 (28.5–29.0)		30.4 (30.0–30.8)		14.8 (14.6–15.0)	
Current alcohol use						
No	28.0 (27.8–28.2)	< 0.001	40.2 (39.8–40.7)	< 0.001	19.9 (19.5–20.3)	< 0.001
Yes	30.0 (29.9–30.2)		34.6 (34.3–34.9)		14.8 (14.6–15.0)	
Exercise						
No	31.3 (30.8–31.8)	< 0.001	31.1 (30.2–32.0)	< 0.001	14.5 (13.8–15.1)	< 0.001
Irregular exercise	31.1 (30.9–31.3)		39.3 (38.9–39.7)		17.9 (17.6–18.3)	
Regular exercise	28.2 (28.0–28.3)		35.1 (34.8–35.5)		15.7 (15.5–16.0)	
Body mass index (kg/m^2^)						
18.50–22.99	17.0 (16.8–17.2)	< 0.001	31.0 (30.5–31.6)	< 0.001	16.7 (16.2–17.1)	< 0.001
< 18.50	12.1 (11.5–12.7)		33.4 (30.9–36.1)		17.3 (15.3–19.5)	
23.00–24.99	26.3 (26.0–26.5)		34.5 (34.0–35.0)		16.8 (16.4–17.2)	
25.00–29.99	37.4 (37.1–37.6)		37.0 (36.7–37.4)		16.2 (15.9–16.5)	
≥ 30.00	53.1 (52.6–53.5)		39.8 (39.2–40.4)		14.7 (14.2–15.1)	

*CI* confidence interval.

### Factors associated with hypertension

Multivariable adjusted odds ratios from the logistic regression model are shown in Table 5. After mutually adjusting for demographic and behavioralcharacteristics, the prevalence of HTN was higher for males than females (AOR 2.04; 95% CI 1.97–2.10); and those with T2D than those without T2D (AOR 5.95; 95% CI 5.81–6.09). Regarding behavioral factors, the prevalence of HTN was higher among RTA personnel who reported current smoking (AOR 1.04; 95% CI 1.03–1.06) and current alcohol consumption (AOR 1.19; 95% CI 1.17–1.21). In addition, a higher BMI was associated with the prevalence of HTN in a dose–response relationship. Finally, those reporting regular exercise had a lower HTN prevalence than sedentary participants (AOR 0.89; 95% CI 0.86–0.91).

**Table 5:** Multivariable analysis for factors associated with prevalence, awareness, and control of hypertension (2017–2021).

Factors	Hypertension		Awareness		Control	
	AOR (95% CI)	p-value	AOR (95% CI)	p-value	AOR (95%CI)	p-value
Sex						
Female	1		1		1	
Male	2.04 (1.97–2.10)	< 0.001	0.70 (0.66–0.74)	< 0.001	0.63 (0.59–0.67)	< 0.001
Age (years)						
< 30	1		1		1	
30–39	1.55 (1.52–1.58)	< 0.001	1.66 (1.56–1.76)	< 0.001	1.38 (1.27–1.49)	< 0.001
40–49	2.66 (2.60–2.72)	< 0.001	3.60 (3.4–3.82)	< 0.001	2.31 (2.14–2.49)	< 0.001
50–59	4.65 (4.55–4.75)	< 0.001	5.70 (5.39–6.04)	< 0.001	2.82 (2.62–3.04)	< 0.001
60	7.66 (7.06–8.31)	< 0.001	6.73 (5.94–7.62)	< 0.001	2.63 (2.31–2.99)	< 0.001
Regions						
Bangkok	1		1		1	
Central	1.70 (1.66–1.74)	< 0.001	0.69 (0.66–0.72)	< 0.001	0.75 (0.72–0.78)	< 0.001
Northeast	2.09 (2.04–2.14)	< 0.001	0.48 (0.46–0.51)	< 0.001	0.59 (0.56–0.62)	< 0.001
North	1.65 (1.60–1.69)	< 0.001	0.55 (0.52–0.58)	< 0.001	0.72 (0.69–0.76)	< 0.001
South	1.55 (1.50–1.60)	< 0.001	0.27 (0.25–0.29)	< 0.001	0.34 (0.31–0.36)	< 0.001
Scheme						
Civil servant medical benefit	1		1		1	
Social Security	1.04 (0.98–1.12)	0.214	1.14 (1.00–1.29)	0.047	0.97 (0.84–1.12)	0.683
Universal Coverage	0.93 (0.85–1.03)	0.166	0.39 (0.32–0.48)	< 0.001	0.54 (0.42–0.69)	< 0.001
History of diabetes						
No	1		1		1	
Yes	5.95 (5.81–6.09)	< 0.001	11.47 (11.12–11.83)	< 0.001	5.28 (5.11–5.45)	< 0.001
Current smoker						
No	1		1		1	
Yes	1.04 (1.03–1.06)	< 0.001	0.93 (0.90–0.96)	< 0.001	0.98 (0.95–1.02)	0.351
Current alcohol use						
No	1		1		1	
Yes	1.19 (1.17–1.21)	< 0.001	0.95 (0.92–0.98)	0.002	0.80 (0.77–0.83)	< 0.001
Exercise						
No	1		1		1	
Irregular exercise	0.97 (0.94–0.99)	0.027	1.47 (1.39–1.56)	< 0.001	1.23 (1.15–1.31)	< 0.001
Regular exercise	0.89 (0.86–0.91)	< 0.001	1.32 (1.25–1.40)	< 0.001	1.14 (1.07–1.21)	< 0.001
Body mass index (kg/m^2^)						
18.50–22.99	1		1		1	
< 18.50	0.80 (0.75–0.86)	< 0.001	1.12 (0.97–1.30)	0.121	1.02 (0.86–1.20)	0.846
23.00–24.99	1.45 (1.42–1.48)	< 0.001	1.03 (0.98–1.07)	0.222	0.89 (0.85–0.93)	< 0.001
25.00–29.99	2.31 (2.27–2.36)	< 0.001	1.09 (1.05–1.14)	< 0.001	0.77 (0.74–0.80)	< 0.001
≥ 30.00	4.86 (4.74–4.99)	< 0.001	1.31 (1.25–1.38)	< 0.001	0.63 (0.59–0.66)	< 0.001
Year						
2017	1		1		1	
2018	1.03 (1.01–1.06)	0.013	1.05 (1.00–1.10)	0.045	1.08 (1.02–1.14)	0.004
2019	0.94 (0.92–0.96)	< 0.001	1.02 (0.98–1.07)	0.285	1.02 (0.97–1.07)	0.461
2020	0.97 (0.94–0.99)	0.003	1.14 (1.09–1.19)	< 0.001	1.15 (1.09–1.21)	< 0.001
2021	1.02 (1.00–1.04)	0.082	1.15 (1.10–1.20)	< 0.001	1.21 (1.15–1.27)	< 0.001

Adjusting for age, sex, regions, health insurance scheme, a history of diabetes, smoking status, alcohol use, exercise, body mass index, and years.

*AOR* adjusted odds ratio, *95% CI* 95% confidence interval.

### Awareness of hypertension

Overall, of RTA personnel with HTN, 35.9% had been aware of their condition for over five years (Table 3). HTN awareness was significantly higher among females (54.9%) than among males (34.8%; *p-value* < 0.001). RTA personnel who were older were more likely to have HTN awareness. According to geographic region, HTN awareness was the lowest among participants residing in the south (20.4%; Table 4).

### Factors associated with awareness of hypertension

After adjusting for demographics and behavioral characteristics, males were less likely to have an awareness of HTN (AOR 0.70; 95% CI 0.66–0.74) and older participants were more likely to have an awareness of HTN, with a dose–response relationship. In addition, awareness of HTN was lower among individuals with current smoker status, with current alcohol consumption, and with no exercise. On the other hand, participants with a BMI $\ge$ 25 kg/m^2^ had a higher prevalence of HTN awareness than those with lower BMI (Table 5).

### Control of hypertension

The overall control of HTN among RTA personnel with HTN was 15.8% in 2017 and remained steady at 17.6% in 2021 (Table 3). Control of HTN among females (30.4%) was better than among males (15.4%; *p-value* < 0.001). Compared with older individuals, younger individuals aged less than 30 years had the lowest control of HTN (4.3%). Regarding geographic region, the control of HTN was the lowest among participants with HTN residing in the south (7.9%; Table 4).

### Factors associated with controlled hypertension

The independent factors associated with controlled HTN are presented in Table 5. After adjusting for demographics and behavioral characteristics, males were less likely to have controlled HTN than females (AOR 0.63; 95% CI 0.59–0.67). Participants aged $\ge$ 30 years were more likely to have controlled HTN than individuals aged less than 30 years. RTA personnel with a history of T2D were more likely to have controlled HTN than those without T2D (AOR 5.28; 95% CI 5.11–5.45). Regarding behavioral factors, individuals with current alcohol consumption were less likely to have controlled HTN than participants who were not current drinkers (AOR 0.80; 95% CI 0.77–0.83), while no association was observed between current smoking and control of HTN. Moreover, RTA personnel who exercised had a significantly higher prevalence of controlled HTN than those with no history of exercise. In addition, compared to the participants with normal weight (BMI 18.5–22.9 kg/m^2^), the individuals with higher BMI were less likely to have controlled HTN.

The results of the sensitivity analysis are presented in Supplementary Table S1. The sensitivity analysis using generalized linear mixed models to include random effects to account for RTA hospital variability revealed that the factors associated with HT prevalence, awareness, and control relatively followed the same pattern as the primary analysis.

## Discussion

The present study constitutes the most extensive epidemiological study of HTN prevalence, awareness, and control among RTA personnel in Thailand. We found that approximately one-third of RTA personnel had HTN. Among the participants with HTN, 35.9% were aware of their condition, and 16.2% had controlled HTN. The awareness of HTN and control rates remained steady over five years. We also found that a higher prevalence of HTN was associated with demographic and behavioral factors, including male sex, older age, residing in the northeast, having a history of T2D, current smoking, current alcohol use, no exercise, and higher BMI. Regarding awareness of HTN, we found that there was still room for improvement among young RTA personnel with HTN, males with HTN and participants residing in the south, as they showed the lowest levels of awareness. Furthermore, a lower awareness of HTN was associated with participants without a history of T2D, current smoking, current alcohol use, no exercise, and lower BMI. Finally, in terms of HTN control, factors associated with a low prevalence of HTN control included male sex, younger age, no history of T2D, current alcohol consumption, no exercise, and higher BMI.

In Thailand, community-based screening for HTN is offered for Thai civilians aged ≥ 35 years. In addition, diagnosis, treatment, and laboratory testing for HTN are also offered to all individuals within the primary health care system^14^. In 2019, 88% of the eligible target population were reported to have been screened for HTN. Nevertheless, most such patients do not report for a follow-up to confirm the diagnosis due to the lack of a systematic follow-up mechanism^14^. In 2013, a related study indicated that only one-third of the diagnosed cases received treatment within six months^24^. The Ministry of Public Health is devising several strategies to improve screening and diagnosis of HTN; for example, improving the accuracy of BP measurement, offering home BP measurement to selected patients, and removing patient barriers to office BP measurement^14^. On the other hand, among RTA personnel, a routine annual health examination is provided at RTA hospitals for RTA personnel who are at least 18 years of age. If increased BP is detected, the individual will be promptly invited to receive proper care at that hospital. Unfortunately, there was limited information on reports for a follow-up to confirm the diagnosis and treatment of HTN. Therefore, this limitation indicated that the assessment of the rate of confirming the diagnosis and treatment should be established to reduce the gap between screening and diagnosis and improve the quality of HTN management for RTA personnel.

According to the NHES V in 2014 and the NHES VI in 2019, the overall HTN prevalence of Thai adults aged at least 15 years rose from 24.7 to 25.4%^10,11^. Similarly, the overall HTN prevalence in the present study increased from 29.3% in 2017 to 30.6% in 2021. However, a higher HTN prevalence in the present study was observed. This finding may be explained by the difference in behavioral factors and higher obesity prevalence among this study population, which was established to be at risk for HTN^25–29^. In general, RTA personnel tended to have a higher prevalence of tobacco use and alcohol consumption than the general Thai population. The present study demonstrated that approximately one-third of RTA personnel were current smokers, while the NHES VI reported that 15.2% of Thai adults reporting currently smoked^11^. Similarly, the prevalence of current alcohol consumption among the Thai population was 44.6%^11^, whereas from 2017 to 2021, 65.4% to 71.2% of RTA personnel reported current alcohol use. We also found that the obesity prevalence among this study population was relatively high when compared with the general Thai population (42.2%), especially in males^11^.

Globally, 59% of women and 49% of men with HTN report a previous diagnosis of hypertension^3^. In Thailand, the NHES VI reported that HTN awareness among Thai patients with HTN was 59.5% among females and 43% among males^11^. Of the RTA personnel with HTN, 35.9% were aware of HTN. This phenomenon may be explained by the institutional structures of the military that lead to detrimental behaviors among RTA personnel and weaken their attempts to embrace healthier habits^30^. In addition, beliefs about masculinity that are embedded in the culture and integrated with social institutions contribute to the behavioral patterns of military personnel in ways that influence health awareness^31^.

Our study found that HTN awareness was significantly greater among females (54.9%) than males (34.8%). The previous study supported sex differences in HTN awareness because of the lower use of health care services among males than females. For example, females regularly interact with healthcare providers for gynecological health; men are not faced with this reason to visit health care professionals^32,33^. Therefore, females have more opportunities to measure and be aware of their BP and HTN status. Therefore, enhancing health literacy and awareness efforts aimed at men (approximately 90% of RTA personnel), for instance, peer-led interventions and incremental community empowerment, may modify cultural institutions to fortify helpful health behaviours among military personnel^31,34,35^. In addition, providing an automatic BP machine at military units for self-BP monitoring may be a tool to increase HTN awareness^36^.

The NHES in Thailand reported that the prevalence of HTN control dropped by 7.1% from 2014 to 2019^11^. Likewise, we also found that HTN control among RTA personnel with HTN was not improved over five years. Most healthcare workers in Thailand, including RTA Hospitals, use the treatment guidelines produced by the Thai Hypertension Society, which provide a menu of options for treatment based on age and comorbidities^18^. However, simple drug- and dose-specific protocols for treatment are not available. In addition, earlier clinical audits had also found that a lack of adherence to treatment guidelines is common, for instance, the use of monotherapy when dual therapy was needed^14^. Therefore, the HTN control rate among RTA personnel was not improved. Regarding the existing evidence^37^, introducing a simple, step-by-step drug- and dose-specific treatment protocol consistent with the national guidelines should be established^14^. In addition, introducing fixed-dose combination pills is also an essential means of improving BP control without increasing the side effects^38,39^.

Health systems barrier may be another reason for the HTN control rate among RTA personnel that was not developed^14^. All RTA personnel are working population who find it inconvenient to attend healthcare facilities during working hours. Therefore, access to HTN care can easily be improved by implementing an out-of-hour clinic for diagnosis, treatment, and monitoring for RTA personnel who cannot leave their working period to visit the doctor.

In line with the literature^4,40–42^, a difference was noted in age and BMI among participants of both sexes. Our multivariable analysis showed that male participants were more likely than female participants to have HTN. Similarly, we also found that males had a higher risk than females for uncontrolled BP, which was similar to related studies in Thailand^12^, the US^43^ and China^44^. The mechanism for gender differences in BP control may be explained by biological factors, including hormonal effects on elevated BP^45,46^. There is significant evidence that testosterone plays an important role in gender-associated differences in BP regulation^47^. Furthermore, related studies demonstrated that pro-renin and renin levels were lower among females than among males, resulting in higher BP among males than females^46,48^.

In terms of age, we found that participants who were older than 30 years had a higher prevalence of HTN than younger participants, with a dose–response relationship, that was consistent with related reports^41,49^. Higher age was associated with incremental increases in BP^50^. This phenomenon could be explained by vascular ageing and degenerative processes, where changes occur in endothelial function, including loss of arterial elasticity as well as arterial stiffness^50,51^. This finding suggests that the high prevalence of HTN among RTA personnel should be recognized, especially among older individuals who are prone to ASCVD. Continued monitoring and treatment adherence and long-term care may play an essential role in preventing further complications^52,53^.

On the other hand, our study revealed low rates of HTN awareness and poor control of HTN among RTA personnel who were young, which is a common observation globally^12,54–56^. Because younger individuals tend to be healthier, they are less likely to regularly visit health care providers, diminishing the possibility that they will know their true BP status^32^. In addition, HTN complications may not arise in the early phase of HTN^57^; therefore, younger individuals with HTN lack awareness of hypertensive complications. Furthermore, younger RTA personnel may have more responsibilities, such as deployment; thus, they may not follow up with medical doctor appointments due to timing^12,13^. Thus, the continuity of care for HTN, i.e., improving awareness and health literacy, continued monitoring and adherence, and lifestyle modifications to improve the risk profile, should be designed to appeal to younger individuals^52,58^.

We found that compared with RTA personnel without T2D, those with existing T2D had a higher HTN prevalence. This observation was likely due to the well-documented positive relationship between T2D and HTN^59,60^. Both HTN and T2D contribute to vascular stiffness and dysfunction, resulting in ASCVD^31,32^. Several studies have indicated that antihypertensive drugs reduce ASCVD among individuals with T2D^63,64^. Therefore, we suggest that more specific programs should be implemented to target individuals with T2D, such as closely monitoring their BP and enhancing their antihypertensive drug adherence^63^.

In line with the literature^65,66^, our findings showed that individuals with T2D tended to have higher HTN awareness than those without T2D. Furthermore, we found that participants with T2D were associated with controlled HTN. The management of HTN in Thailand may explain this phenomenon. Thai guidelines on treating hypertension have been released to all physicians, including those in RTA hospitals^18^. Therefore, patients with HTN have been receiving appropriate treatment, including antihypertensive drugs and patient education, especially among patients with HTN and T2D, which may have suggested tight control of BP.

Our findings reported that demographic and behavioral factors were associated with the prevalence, awareness, and control of HTN. We observed that HTN prevalence among RTA personnel residing in the northeast was the highest compared with other regions in Thailand over five years. The NHES VI reported that the HTN prevalence in the northeast was relatively low compared with that in other regions^11^. These results emphasized that the HTN burden in this study population differed from that of the general Thai population. However, the NHES VI also reported that the general Thai population residing in the northeast had the lowest obesity prevalence (39%) when compared with those in other regions^11^.

The present study reported that HTN prevalence among current smokers was more likely to be higher than that among those who did not currently smoke. This finding was consistent with multiple related lines of evidence^25,26,67^, indicating that rising BP was significantly related to current smoking status. Smoking can affect BP in acute phases and contributes to pathological conditions through the direct effect of nicotine on the peripheral sympathetic ganglia and neuromuscular junction^68–70^. We also observed that a lack of awareness of HTN was associated with current smoking status; moreover, the prevalence of current smoking among RTA personnel continuously increased from 28.5% in 2017 to 33.4% in 2021^11^. Compared with the NHES VI, our findings indicated that the prevalence of current smoking among RTA personnel was higher than that among the general Thai population (15.2%). The stress and tension due to the workload of military personnel could be the factors underlying the higher rates of smoking, which is an effort to relax and relieve stress; moreover, smoking is also said to help military personnel stay alert^71–73^. Our findings emphasized that smoking is an essential health issue in this population. Therefore, smoking cessation, which is the primary prevention for HTN, should be encouraged in this population. Enlisting community leaders, such as commandants, to discourage cigarette smoking and promote cessation may likely yield success^74^. Advice to stop smoking from health care personnel during annual physical health examinations and tobacco cessation support should be provided^75^. The evidence reported that pharmacotherapy contributes to successful smoking cessation^76^; in Thailand, the UHC covers this benefit package; therefore, it would be feasible that everyone willing to quit tobacco use could access this service.

Like smoking behavior, the prevalence of current alcohol consumption was higher among RTA personnel than among the general Thai population (32.5%)^11^. Alcohol and tobacco seem to go together. Several studies have demonstrated a positive relationship between alcohol consumption and smoking^77,78^. Generally, there has been a strong tradition of alcohol consumption among military personnel; moderate consumption may be a crucial catalyst for bonding and cohesion in the military^79,80^. Excessive alcohol consumption is a known behavioral risk factor for HTN^27,81^. This study found that participants reporting current alcohol consumption were more likely to present HTN than abstainers. Furthermore, our results indicated that a lack of awareness of HTN and uncontrolled HTN were associated with current alcohol consumption. Consistent evidence demonstrated the effect of alcohol on increased BP through several pathways, including enhanced sympathetic activity, the stimulation of the renin–angiotensin–aldosterone system, increased vascular reactivity and increased arterial stiffness^81,82^.

Our findings suggested that reduced alcohol consumption should be encouraged among RTA personnel. The meta-analysis of trial data demonstrated that reducing alcohol consumption among individuals who consumed more than two drinks daily could significantly reduce SBP and DBP^83^. According to the cultural context among military personnel, reduced harmful use of alcohol may be a priority^84^. Therefore, a pattern of alcohol consumption should be assessed in annual physical health examinations; then, motivational interventions such as a Brief Negotiated Interview to encourage clients to change their risky behaviors should be provided^85^.

We observed that higher BMI was positively correlated with rising SBP and DBP. Furthermore, the prevalence of HTN and uncontrolled HTN was higher among participants with higher BMI than among those with normal BMI (18.50–22.99 kg/m^2^), with a dose–response relationship, which was compatible with a related study in Thailand^12^. Individuals with obesity have higher adiposity levels, which are the substrate for producing leptin; this results in increased leptin levels and heightened sympathetic nerve activity, which is associated with elevated BP^28,86^. Additionally, a recent study revealed the continuously rising proportion of RTA personnel with a BMI $\ge$ 25 kg/m^2^^16^. Obviously, obesity is an essential health issue in this population and is associated with a higher prevalence of HTN as well as poor control of BP. The initiation of a weight management program via annual physical health examinations should be established, such as informing and enhancing awareness of body weight and BMI. Furthermore, unhealthy dietary behavior is a crucial component that should be effectively managed^87^. In addition, multimodality interventions may be used, for instance, calorie restriction^88^, daily self-weighing^89^, and a support system using a chatbot or human resources^90^.

Our findings reported that regular exercise was associated with a lower HTN prevalence among RTA personnel. Therefore, our study suggested that weight management as regular exercise should be encouraged to decrease BMI, lower the prevalence of HTN and attenuate the risk for ASCVD^91,92^. Annually, a physical fitness test is conducted by military units nationwide. However, vigorous physical activity can rarely trigger heat-related illness or acute cardiovascular events^93,94^, so structured exercise should be performed based on relevant standard guidelines^95^. Additionally, physical activity may be enhanced by enforcing that RTA personnel must meet a minimum physical activity requirement each day, such as a step count of 10,000 steps daily or 70,000 steps weekly^96^. This physical activity may be monitored by a pedometer or mobile application, which is available and feasible^97^. In addition, if RTA personnel cannot meet the minimum requirement, the monitoring system may involve a chatbot that will provide feedback on their level of physical activity for improvement^98^.

Several limitations were encountered in this study. First, the study employed a serial cross-sectional design; therefore, causality cannot be inferred. Second, in the present study, HTN treatment was not reported because the question regarding the comorbidity of HTN in the physical health examinations was relatively crude: “Have you been diagnosed with HTN or taken antihypertensive drugs?” A response of “Yes” means that (1) an individual has been diagnosed with HTN, (2) he or she takes antihypertensive drugs or (3) he or she has been diagnosed with HTN and takes antihypertensive drugs. Therefore, we cannot mutually distinguish the component of HTN treatment from the diagnosis. However, this limitation indicated that the assessment of HTN treatment among RTA personnel in routine physical health examinations should be improved to obtain essential information for improving HTN management. Third, according to the nature of an observational study, data on some variables were missing, including smoking status (2.4%), alcohol consumption (2.0%) and exercise (2.8%). Although we were aware of missing data, the present study consisted of an extensive sample size to include the existing data in the analysis, and the associations between factors and outcomes were able to be presented with relatively precise results. Fourth, this study was conducted among a sample of RTA personnel consisting of a higher proportion of males; moreover, the demographic characteristics of this population differ from those of the Thai civilian population, leading to a limited contribution to the ultimate implication of Thailand's public health policy. However, the results reported the actual situation in this population. Fifth, because we used the collected data, the information on family history, socioeconomic status, total calorie intake, and nutritional status were not included in the analysis; thus, the residual confounding on the association between outcomes and exposure may have existed. Next, this study covered the period of the COVID-19 pandemic, especially in 2020 and 2021: the nationally imposed lockdown measure may have affected some behaviors of RTA personnel; however, the variable of year was adjusted in the multivariable analysis to determine the factors associated with the outcome. Finally, due to the use of secondary data, some variables were collected broadly, e.g., the intensity and frequency of alcohol consumption, tobacco use, and exercise were not recorded. However, the associations between behavioral factors and interesting outcomes could be demonstrated.

The present study also exhibited significant strengths, including a representative, large sample of RTA personnel. Thus, our findings provide valuable insights regarding HTN prevalence, awareness and control in the military population in Thailand. Furthermore, these data may contribute to strategies for the primary prevention of HTN among RTA personnel.

## Conclusion

Our study emphasized that HTN was a common health issue in this population, especially among male RTA personnel and older individuals. Obesity and behavioral risk factors are potential targets for intervention to attenuate the risk for HTN and improve control of HTN. Awareness of HTN and control of HTN among male participants and younger individuals should be improved. Furthermore, behavioral risks, including alcohol consumption, tobacco use, and sedentary behavior, which were associated with lower HTN awareness, should be managed simultaneously.

## Supplementary Information

Supplementary Information.

## Data Availability

The data that support the findings of this study are available from the Royal Thai Army Medical Department, Bangkok, Thailand but restrictions apply to the availability of these data, which were used under license for the current study, and so are not publicly available. Data are, however, available from the authors upon reasonable request and with permission of the Royal Thai Army Medical Department, Bangkok, Thailand (contact Boonsub Sakboonyarat via boonsub1991@pcm.ac.th).

## Abbreviations

HTN: Hypertension
NCDs: Noncommunicable diseases
T2D: Type 2 diabetes
ASCVD: Atherosclerotic cardiovascular diseases
BMI: Body mass index
RTA: The Royal Thai Army
RTAMED: The Royal Thai Army Medical Department
NHES: National Health Examination Survey
AOR: Adjusted odds ratio
CI: Confidence interval
SD: Standard deviation
