# Supplementary material for: Prevalence, awareness, and control of hypertension and associated factors among Royal Thai Army personnel in Thailand from 2017 to 2021
Source: Sci Rep. 2023 Apr 28;13:6946. doi: 10.1038/s41598-023-34023-z (PMC10141845; doi:10.1038/s41598-023-34023-z)
Supplement: Supplementary file 1 — Supplementary Information. [file 41598_2023_34023_MOESM1_ESM.pdf]

Prevalence, Awareness, and Control of Hypertension and Associated Factors among Royal Thai Army Personnel in Thailand from 2017 to 2021

Boonsub Sakboonyarat[1], Jaturon Poovieng[2], Phutsapong Srisawat[1], Panadda Hatthachote[3], Mathirut Mungthin[4], Ram Rangsin[1], *Kanlaya Jongcherdchootrakul[1]

[1]Department of Military and Community Medicine, Phramongkutklao College of Medicine, Bangkok, Thailand 10400

[2]Department of Medicine, Phramongkutklao College of Medicine, Bangkok, Thailand 10400

[3]Department of Physiology, Phramongkutklao College of Medicine, Bangkok, Thailand 10400

[4]Department of Parasitology, Phramongkutklao College of Medicine, Bangkok, Thailand 10400

Supplementary Table 1. General linear mixed model analysis for factors associated with Prevalence, Awareness, and Control of Hypertension (2017-2021)

Factors	Hypertension		Awareness		Control	
	AOR (95% CI)	*p* value	AOR (95% CI)	*p* value	AOR (95% CI)	*p value*
**Sex**						
Female	1		1		1	
Male	2.30 (2.21-2.39)	<0.001	0.8 (0.75-0.87)	<0.001	0.58 (0.54-0.63)	<0.001
**Age (years)**						
<30	1		1		1	
30-39	1.55 (1.52-1.58)	<0.001	1.71 (1.61-1.83)	<0.001	1.36 (1.25-1.48)	<0.001
40-49	2.66 (2.60-2.72)	<0.001	3.79 (3.56-4.04)	<0.001	2.34 (2.16-2.53)	<0.001
50-59	4.53 (4.43-4.63)	<0.001	6.00 (5.65-6.37)	<0.001	2.85 (2.63-3.08)	<0.001
60	8.14 (7.31-9.07)	<0.001	7.35 (6.29-8.60)	<0.001	2.89 (2.46-3.41)	<0.001
**Regions**						
Bangkok	1		1		1	
Central	1.73 (1.11-2.71)	0.016	0.58 (0.11-3.21)	0.536	0.48 (0.12-1.97)	0.310
Northeast	1.89 (1.20-2.98)	0.006	0.61 (0.11-3.38)	0.569	0.44 (0.11-1.82)	0.258
North	1.97 (1.25-3.09)	0.003	0.59 (0.11-3.27)	0.544	0.49 (0.12-2.04)	0.329
South	1.59 (0.98-2.58)	0.063	0.18 (0.03-1.14)	0.068	0.18 (0.04-0.86)	0.031
**Health insurance scheme**						
Civil servant medical benefit	1		1		1	
Social Security	1.04 (0.97-1.12)	0.246	1.23 (1.06-1.41)	0.005	1.06 (0.91-1.24)	0.465
Universal Coverage	1.39 (1.23-1.58)	<0.001	0.61 (0.45-0.81)	0.001	0.74 (0.53-1.04)	0.084
**History of diabetes**						
No	1		1		1	
Yes	5.34 (5.2-5.48)	<0.001	11.81 (11.4-12.24)	<0.001	5.23 (5.04-5.42)	<0.001
**Current smoker**						
No	1		1		1	
Yes	1.08 (1.06-1.1)	<0.001	0.87 (0.84-0.9)	<0.001	0.91 (0.88-0.95)	<0.001
**Current alcohol use**						
No	1		1		1	
Yes	1.20 (1.18-1.22)	<0.001	0.81 (0.78-0.84)	<0.001	0.73 (0.7-0.76)	<0.001
**Exercise**						
No	1		1		1	
Irregular exercise	1.02 (0.99-1.05)	0.251	1.2 (1.12-1.28)	<0.001	1.13 (1.04-1.21)	0.002
Regular exercise	0.94 (0.91-0.97)	<0.001	1.1 (1.03-1.17)	0.004	1.06 (0.99-1.14)	0.118
**Body mass index (kg/m^2)**						
18.50-22.99	1		1		1	
<18.50	0.83 (0.77-0.88)	<0.001	1.13 (0.97-1.32)	0.123	1.02 (0.86-1.22)	0.790
23.00-24.99	1.46 (1.43-1.49)	<0.001	1.04 (0.99-1.09)	0.141	0.90 (0.85-0.95)	<0.001
25.00-29.99	2.35 (2.31-2.4)	<0.001	1.14 (1.09-1.19)	<0.001	0.78 (0.74-0.81)	<0.001
30.00-34.99	5.02 (4.88-5.15)	<0.001	1.38 (1.31-1.45)	<0.001	0.65 (0.61-0.69)	<0.001
**Year**						
2017	1		1		1	
2018	1.13 (1.10-1.15)	<0.001	0.97 (0.92-1.02)	0.178	0.94 (0.88-0.99)	0.026
2019	0.99 (0.97-1.02)	0.486	0.98 (0.93-1.03)	0.349	0.89 (0.84-0.94)	<0.001
2020	1.04 (1.01-1.06)	0.007	1.03 (0.98-1.09)	0.193	0.97 (0.92-1.03)	0.305
2021	1.06 (1.03-1.09)	<0.001	0.99 (0.94-1.04)	0.570	1.01 (0.95-1.06)	0.839
**ICC hospital**	0.03 (0.02-0.04)		0.28 (0.19-0.28)		0.21 (0.14-0.30)	

Generalized linear mixed models with a logit link to adjust for age, sex, region, health insurance scheme, a history of diabetes, smoking status, alcohol use, exercise, BMI, and years and including random effects to account for RTA hospital variability.
AOR: adjusted odds ratio, CI: confidence interval
